# Astragalus Mongholicus Polysaccharides Alleviate Kidney Injury in Rats with Type 2 Diabetes Through Modulation of Oxidation, Inflammation, and Gut Microbiota

**DOI:** 10.3390/ijms26041470

**Published:** 2025-02-10

**Authors:** Guoquan Xu, Haisheng Yuan, Jingran Liu, Xianjue Wang, Li Ma, Yuzhen Wang, Guicheng Dong

**Affiliations:** College of Life Sciences, Inner Mongolia Agricultural University, Hohhot 010018, China; xgq117735@163.com (G.X.); yuanhaisheng1226@163.com (H.Y.); liujingran1983@163.com (J.L.); xianjuewang@163.com (X.W.); supermaryfly@126.com (L.M.)

**Keywords:** DN, *Astragalus mongholicus* polysaccharides, oxidative stress, inflammation, gut microbiota

## Abstract

We aimed to uncover the underlying mechanisms contributing to the therapeutic efficacy of *Astragalus mongholicus* Polysaccharides (mAPS) in alleviating diabetic nephropathy (DN). The rat model of DN was subjected to a high-sugar and high-fat diet (HSHFD) coupled with streptozotocin (STZ) injection. Our findings revealed that mAPS administration decreased fasting blood glucose (FBG), BUN, SCR, UA, and MDA levels, while elevating serum GSH, GSH-PX, and SOD activities in DN rats (*p* < 0.05). Furthermore, there was a notable rise in the mRNA and protein expression of renal Nrf-2, GCLC, NQO1, and HO-1 post mAPS treatment (*p* < 0.05). Additionally, mAPS supplementation led to reduced protein expression of TLR4, NLRP3, p-NF-κB, TGF-β, and Smad4. Concurrently, mAPS exerted a modulatory effect on gut microbiota, as evidenced by the increased abundance of *Muribaculaceae*, *Ruminococcus_1*, *Phascolarctobacterium*, and *Lachnoclostridium*-related genera. Spearman correlation analysis illustrated a negative association between the abundance of microbiota (*Muribaculaceae*, *Lachnospiraceae_NK4A136*, *Ruminococcus_1*, *Clostridiales*) and the levels of serum parameters (BUN, CR, UA, TC, TG). In summary, our data robustly attests to the potential of mAPS in modulating oxidative stress, inflammation, and gut microbiota, ultimately resulting in improved renal function in DN rats.

## 1. Introduction

Type 2 diabetes mellitus (T2DM) is a chronic metabolic disease characterized by hyperglycemia due to impaired pancreatic β-cell function and insulin resistance. T2DM is among the most prevalent and rapidly escalating diseases globally. Prolonged exposure to hyperglycemic conditions results in chronic damage to various organs, including the kidneys. Diabetic nephropathy (DN), a common microvascular complication during the progression of T2DM, has emerged as the leading cause of end-stage renal disease (ESRD) [1,2].

The complex pathogenesis of DN requires further investigation. It has been determined that abnormal glucose metabolism, the renal angiotensin system, inflammation, and oxidative stress are closely related to the development of DN [3,4,5]. It is worth noticing that hyperglycemia produces excessive reactive oxygen species (ROS), which in turn exacerbates the kidney’s inflammatory responses, resulting in kidney fibrosis and kidney dysfunction [6].

Evidence indicates that an imbalance in the gut microbiota contributes to the progression of DN [7,8]. The gut-kidney axis theory provides new perspectives on the relationship between gut microbiota and various kidney diseases [9]. Meanwhile, probiotic treatments (e.g., *Bifidobacterium longum* subsp., *Lactobacillus acidophilus*, and *Bifidobacterium bifidum*) [10,11,12] and dietary interventions (e.g., Cordyceps cicadae polysaccharides and Bupleurum polysaccharides) [13,14] have proven successful in treating and mitigating DN. Thus, influencing the gut microbiota and dietary interventions are an effective way of treating DN.

At present, the clinical management of DN involves sodium glucose cotransporter 2 inhibitors [15], angiotensin converting enzyme inhibitors [16], and GLP-1 receptor agonists [17]. However, the long-term efficacy of these drugs is still unsatisfactory [18,19]. This highlights an urgent need to understand the pathogenesis of DN and to develop novel therapeutic agents. Given their safety, efficacy, and beneficial effects in treating diabetic nephropathy, many natural traditional medicines are considered acceptable alternative treatments. Among these is Shenqi Jiangtang Granules, a Chinese herbal formula documented in the World of Traditional Chinese Medicine (WTCM) and used in the clinical treatment of T2DM [20]. *Astragalus membranaceus* (Fisch.) Bge. var. *mongolicus* (Bge.) Hsiao or *Astragalus membranaceus* (Fisch.) Bge, a key component of this formula, is commonly utilized as a dietary supplement for managing T2DM.

*Astragalus mongholicus* polysaccharides (mAPS) are important bioactive components in *Astragalus mongholicus* Bunge. Previous studies have shown that mAPS possess various pharmacological properties, including immunomodulatory [21], anti-inflammatory [22], and antioxidant [23] effects. Our previous study demonstrated that mAPS has the capacity to alter gut microbiota, manage hepatic glycolipid metabolism, and mitigate inflammation, thereby improving insulin resistance in diabetic rats [24]. However, the comprehensive impacts and mechanisms of mAPS in combating DN remain unexplored. In this study, a DN rat model was established by administering a low dose of Streptozotocin (STZ) combined with a high-sugar and high-fat diet (HSHFD). Subsequently, the effects of mAPS on oxidative stress, inflammation, and gut microbiota in DN rats were examined.

## 2. Results

### 2.1. mAPS Supplementation Affects the General Changes in HSHFD/STZ-Induced Diabetic Rats

**Figure 1:**
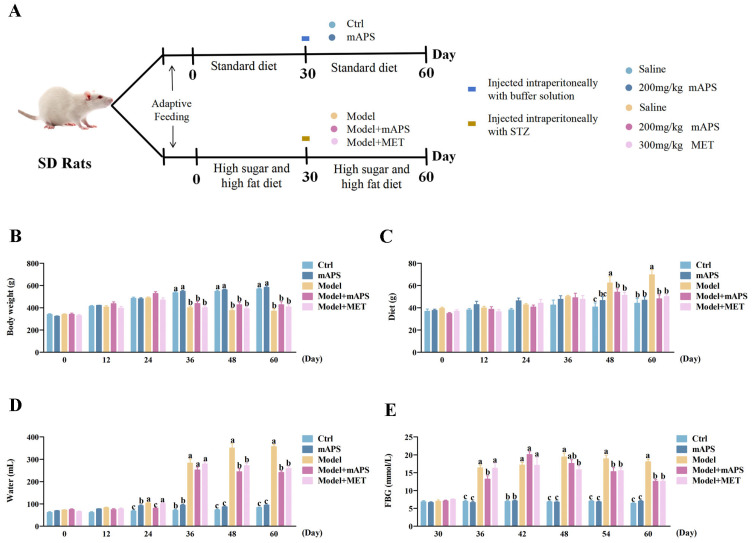
mAPS supplementation affects the general changes in HSHFD/STZ-induced diabetic rats (*n* = 5). (**A**) Route of the experimental schema. (**B**) The change of body weight. (**C**) The change of diet. (**D**) The change of water drinking. (**E**) The change of serum FBG. Data are expressed as mean ± SD. Data with different letters are significantly different (*p* < 0.05).

The experimental design and animal grouping are shown in Figure 1A. In this work, rats in the model group showed clinical T2DM symptoms of polydipsia, polyphagia, polyuria, and weight loss. As shown in Figure 1B, a significant reduction in body weight after STZ injection was found in the Model group compared to the Control group (*p* < 0.05); there was no significant change in weight after mAPS supplementation. Figure 1C,D shows that from day 48 onwards, the Model group exhibited significantly higher diet and water intake compared to the Control group (*p* < 0.05). After treatment with mAPS and MET, both diet and water intake significantly decreased compared to the Model group (*p* < 0.05). The FBG levels of the model rats abnormally increased from 36 days, which was much higher than that in the Control group (Figure 1E, *p* < 0.05). However, FBG levels in the Model + mAPS and Model + MET groups were significantly reduced compared to the Model group (Figure 1E, *p* < 0.05).

### 2.2. mAPS Ameliorated Renal Injury in HSHFD/STZ-Induced Diabetic Rats

**Figure 2:**
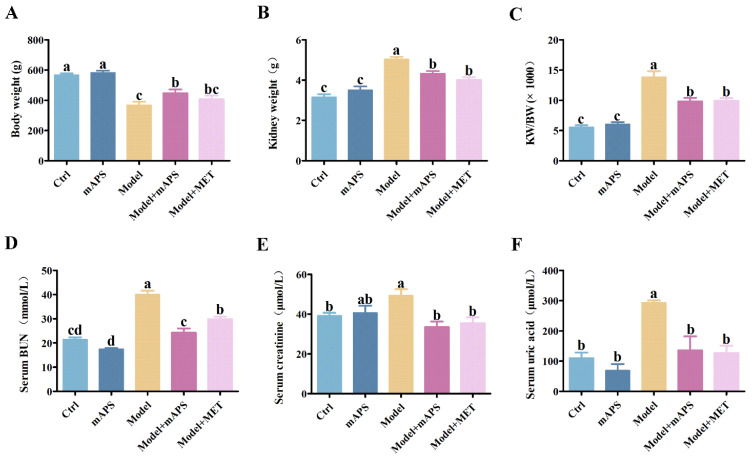
mAPS treatment improved the renal function of the HSHFD/STZ-induced diabetic rats. (*n* = 5). (**A**) The change of body weight. (**B**) The change of kidney weight. (**C**) The alteration in the ratios of kidney weight (KW) to body weight (BW). (**D**) The change of serum BUN. (**E**) The change of serum CR. (**F**) The change of serum UA. Data are expressed as mean ± SD. Data with different letters are significantly different (*p* < 0.05).

To assess the protective effect of mAPS against HSHFD/STZ-induced DN, several biochemical markers related to renal function were measured, including the kidney weight/body weight (KW/BW), blood urea nitrogen (BUN), creatinine (CR), and uric acid (UA). Our findings indicated that the levels of these biochemical indices of the Model group were notably higher than those of the Control group (Figure 2A–F, *p* < 0.05). However, after 30 days of treatment with mAPS, the levels of these renal biomarkers were significantly decreased compared to those in the Model group (*p* < 0.05).

### 2.3. mAPS Ameliorated Dyslipidemia in HSHFD/STZ-Induced Diabetic Rats

**Figure 3:**
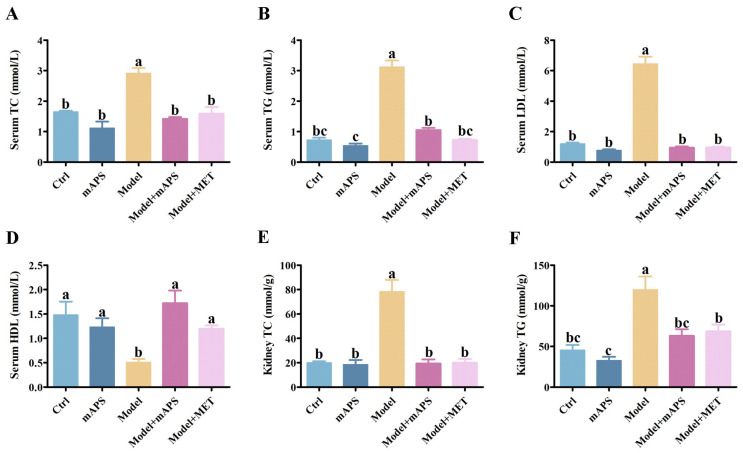
mAPS treatment ameliorated dyslipidemia in HSHFD/STZ-induced diabetic rats (*n* = 5). (**A**) The change of serum TC. (**B**) The change of serum TG. (**C**) The change of serum LDL. (**D**) The change of serum HDL. (**E**) The change of renal TC. (**F**) The change of renal TG. Data are expressed as mean ± SD. Data with different letters are significantly different (*p* < 0.05).

The Model group exhibited significantly elevated serum levels of triglycerides (TG), total cholesterol (TC), and low-density lipoprotein (LDL), whereas high-density lipoprotein (HDL) levels were significantly decreased compared to the Control group. However, the administration of mAPS significantly alleviated the above alterations (Figure 3A–D, *p* < 0.05). Furthermore, the concentrations of renal TC and TG were significantly increased in the model rats compared to the control rats. However, Supplementation with mAPS and MET significantly reduced the renal TC and TG levels (Figure 3E,F, *p* < 0.05).

### 2.4. Effect of mAPS on Colon and Kidney Pathological Injury

**Figure 4:**
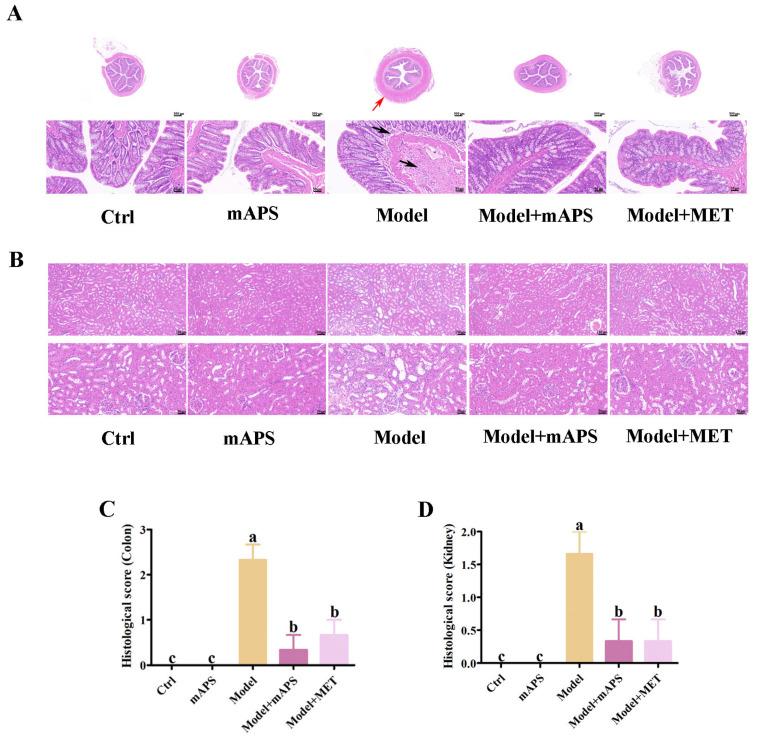
Effects of mAPS treatment on colon and renal histopathology changes (*n* = 3). (**A**) Representative histological images of colon HE staining (The original magnifications used were 20× and 200×, with scale bars representing 500 μm and 50 μm, respectively). (Red arrow: thickening of the muscle layer. Black arrows: lymphocytic infiltration.) (**B**) Representative histological images of renal HE staining (The original magnifications used were 100× and 200×, with scale bars representing 100 μm and 50 μm, respectively) (**C**) Histological score (colon). (**D**) Histological score (kidney). Data are expressed as mean ± SD. Data with different letters are significantly different (*p* < 0.05).

As illustrated in Figure 4A, the colonic fold structure in both the Control and mAPS groups was clear, with an intact mucosal intestinal epithelium and normal morphology of the epithelial cells. In the Model group, there was a thickening of the muscle layer (indicated by the red arrow). Additionally, lymphocytic infiltration was observed at the base of the lamina propria and submucosa in the colons of the Model group (indicated by black arrows). However, supplementation with mAPS led to a reduction in inflammatory cell infiltration and alleviated colon lesions. No notable inflammation was observed in the MET group. HE staining of the kidney revealed that the model rats exhibited diffuse expansion of the mesangial matrix, thickening of the glomerular basement membrane, and a decrease in the capillary lumen (Figure 4B). However, treatment with mAPS attenuated renal structural damage, resulting in significant improvements in mesangial cell hyperplasia and the thickness of the glomerular basement membrane. Based on the aforementioned results, coupled with the histological score (Figure 4C,D), we conclude that mAPS exhibits a certain alleviating effect on colon and kidney lesions.

### 2.5. mAPS Mitigated Oxidative Stress in HSHFD/STZ-Induced Diabetic Rats

**Figure 5:**
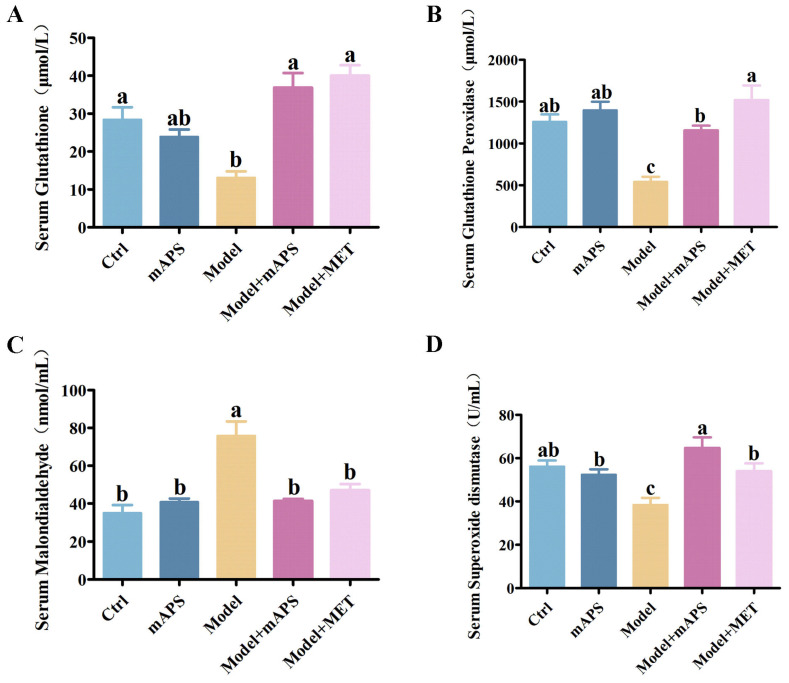
Effects of mAPS treatment on oxidative biomarkers (*n* = 5). (**A**) The change of serum GSH. (**B**) The change of serum GSH-PX. (**C**) The change of serum MDA. (**D**) The change of serum SOD. Data are expressed as mean ± SD. Data with different letters are significantly different (*p* < 0.05).

Oxidative stress plays a pivotal role in the pathological progression of DN. To determine whether mAPS alleviates oxidative stress in model rats, we measured biomarkers related to oxidative stress. As shown in Figure 5A–D, when compared to the Control group, the Model group demonstrated a significant decrease in the activities of glutathione (GSH), glutathione peroxidase (GSH-PX), and superoxide dismutase (SOD), accompanied by a significant increase in the levels of malondialdehyde (MDA) (*p* < 0.05), suggesting the presence of oxidative stress in the model rats. However, mAPS and MET supplementation reversed these alterations in the model rats (*p* < 0.05).

### 2.6. mAPS Activated the Renal Nrf-2/Keap1 Signaling Pathway in HSHFD/STZ-Induced Diabetic Rats

**Figure 6:**
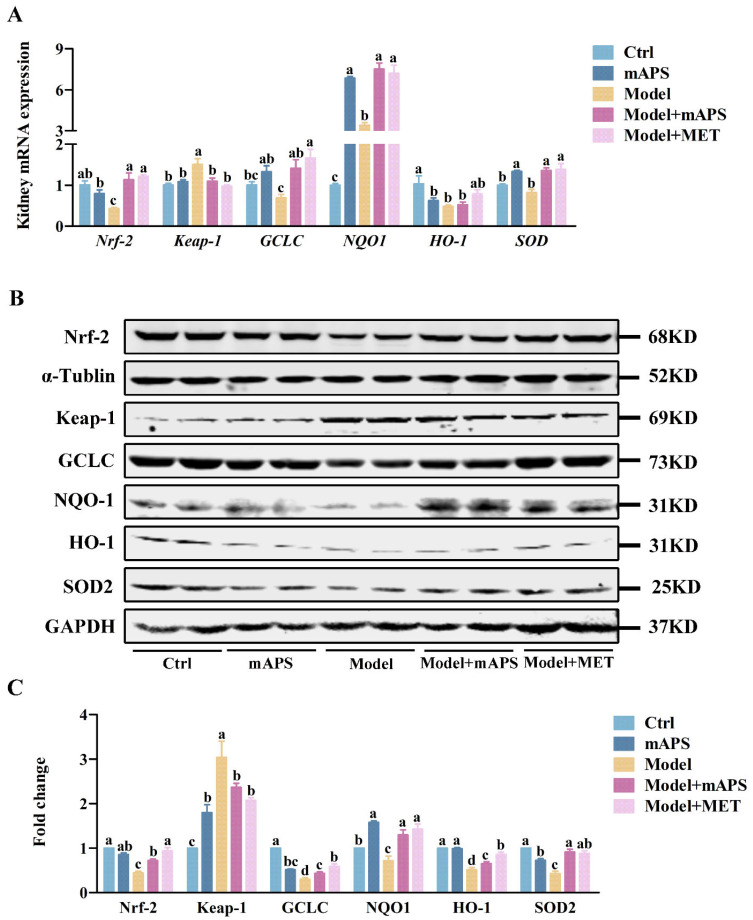
The effect of mAPS on the Nrf-2/HO-1 pathway in the kidney. (**A**) The mRNA expression of *Nrf-2*, *Keap-1*, *GCLC*, *NQO1*, *HO-1*, and *SOD* in kidney. (**B**) The protein expression of Nrf-2, Keap-1, GCLC, NQO1, HO-1, and SOD2 in kidney. (**C**) The fold change of Nrf-2, Keap-1, GCLC, NQO1, HO-1, and SOD2. Data are expressed as mean ± SD. Data with different letters are significantly different (*p* < 0.05).

Next, the expression of genes involved in the classic antioxidant Nrf-2/Keap1 signaling pathway was examined. Quantitative PCR results indicated that the renal mRNA expression levels of Nrf-2 and its downstream targets GCLC, NQO1, HO-1, and SOD were decreased, whereas the expression of Keap-1 mRNA was increased in the Model group compared to the Control group (Figure 6A, *p* < 0.05). However, these changes were reversed after mAPS administration (Figure 6A, *p* < 0.05). To uncover the potential antioxidant mechanism of mAPS, we conducted further analysis of the protein expression of Nrf-2 and its downstream proteins. Western blotting results concurred with those from quantitative PCR, revealing a notable decrease in the protein expression of nuclear Nrf-2 and cytoplasmic GCLC, NQO-1, HO-1, and SOD2, as well as an elevation in renal Keap-1 protein levels in the model rats when compared to the control rats. However, both mAPS and MET treatment significantly reversed these alterations (Figure 6B, *p* < 0.05).

### 2.7. mAPS Inhibits Kidney TLR4/NF-κB Signaling Pathway in HSHFD/STZ-Induced Diabetic Rats

**Figure 7:**
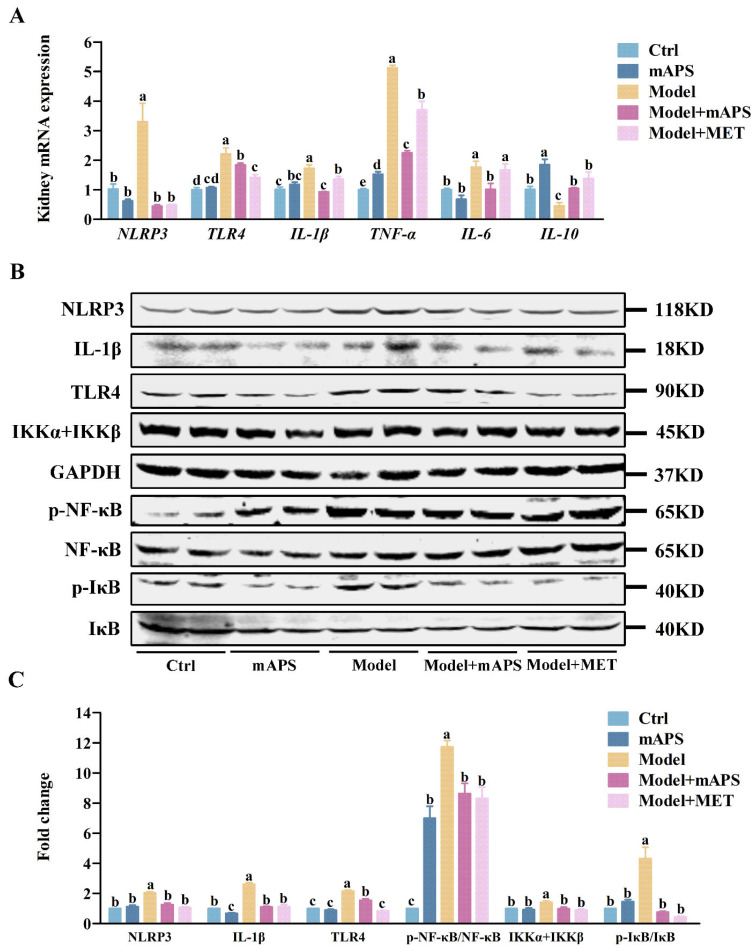
The effect of mAPS on the TLR4/NF-κB pathway in the kidney. (**A**) The mRNA expression of *NLRP3*, *TLR4*, *IL-1β*, *TNF-α*, *IL-10*, and *IL-6* in kidney. (**B**) The protein expression of NLRP3, IL-1β, TLR4, p-NF-κB, IKKα + IKKβ, and p-IκB in the kidney. (**C**) The fold change of NLRP3, IL-1β, TLR4, p-NF-κB, IKKα + IKKβ, and p-IκB. Data are expressed as mean ± SD. Data with different letters are significantly different (*p* < 0.05).

As shown in Figure 7A, the Model group exhibited a notable increase in renal *NLRP3*, *TLR4*, *IL-1β*, *TNF-α*, and *IL-6* mRNA, accompanied by a decrease in *IL-10* mRNA expression when compared to the Control group (*p* < 0.05). Treatment with mAPS and MET significantly lowered the expression of renal *TNF-α*, *IL-1β*, and *IL-6 mRNA* while concurrently increasing the mRNA levels of *IL-10*. To better understand the anti-inflammatory mechanism of mAPS, the renal TLR4/NF-κB pathway was analyzed using Western blotting. The results showed that the renal protein levels of NLRP3, IL-1β, TLR4, p-NF-κB, p-IκB, and IKKα + IKKβ were notably enhanced in the model rats compared to the control rats, while mAPS markedly reversed these outcomes (Figure 7B, *p* < 0.05).

### 2.8. mAPS Improved the Renal Fibrosis in HSHFD/STZ-Induced Diabetic Rats

**Figure 8:**
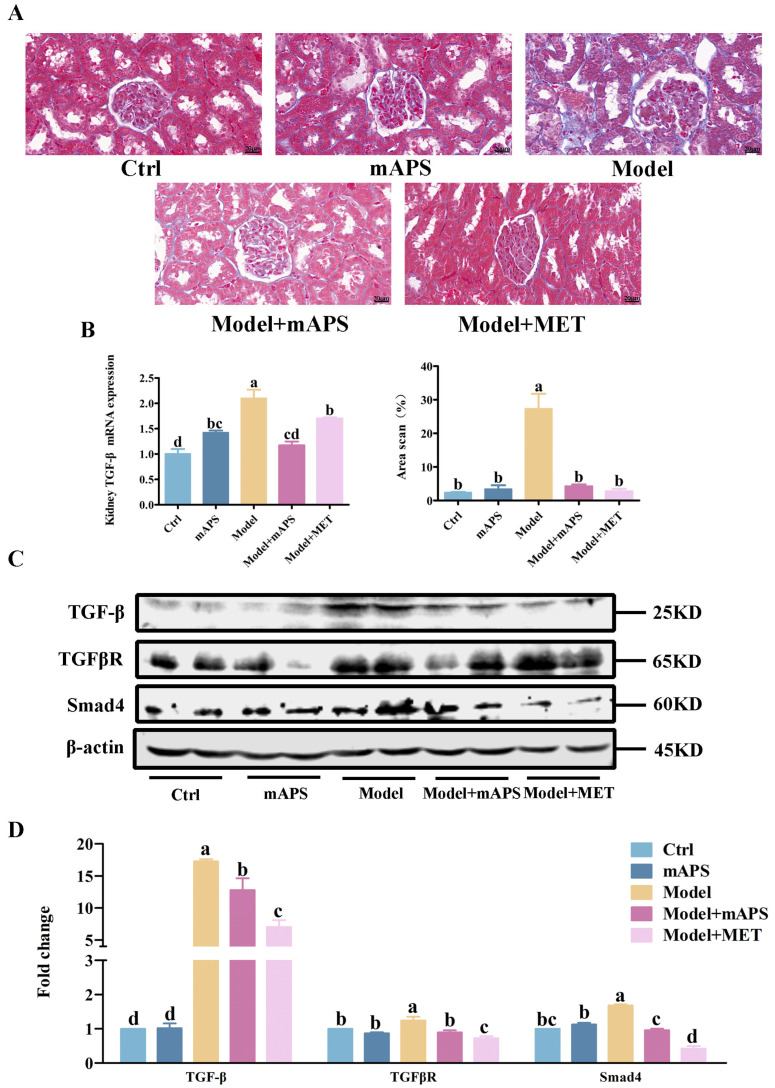
mAPS alleviated renal fibrosis in the diabetic rats (*n* = 3). (**A**) Masson staining analysis (The original magnification is 400×, bar = 20 μm). (**B**) The mRNA expression of *TGF-β* in the kidney. (**C**) The protein expression of TGF-β, TGFβR, and Smad4 in the kidney. (**D**) The fold change of TGF-β, TGFβR, and Smad4. Data are expressed as mean ± SD. Data with different letters are significantly different (*p* < 0.05).

Renal histologic sections were evaluated using the Masson staining technique. Figure 8A depicts that the Model group exhibited prominent renal tubular vacuolization. Additionally, collagen accumulation was evident, as indicated by an expanded blue staining area, suggesting that the kidneys of the model rats underwent fibrosis. In contrast, mAPS treatment led to a relatively normal renal tissue structure, with a notable reduction in the blue-stained area of the interstitium, demonstrating a protective effect.

Furthermore, Western blotting was applied to determine the influence of mAPS on the expression levels of significant proteins in the TGF-β/Smad signaling pathway. Figure 8C indicates that the renal protein expressions of TGF-β, TGFβR, and Smad4 in the Model group were significantly higher than those in the Control group. (*p* < 0.05). mAPS supplementation significantly reduced the renal protein expressions of TGF-β, TGFβR, and Smad4 in the model rats (Figure 8C, *p* < 0.05).

### 2.9. mAPS Reshaped Gut Microbiota in HSHFD/STZ-Induced Diabetic Rats

**Figure 9:**
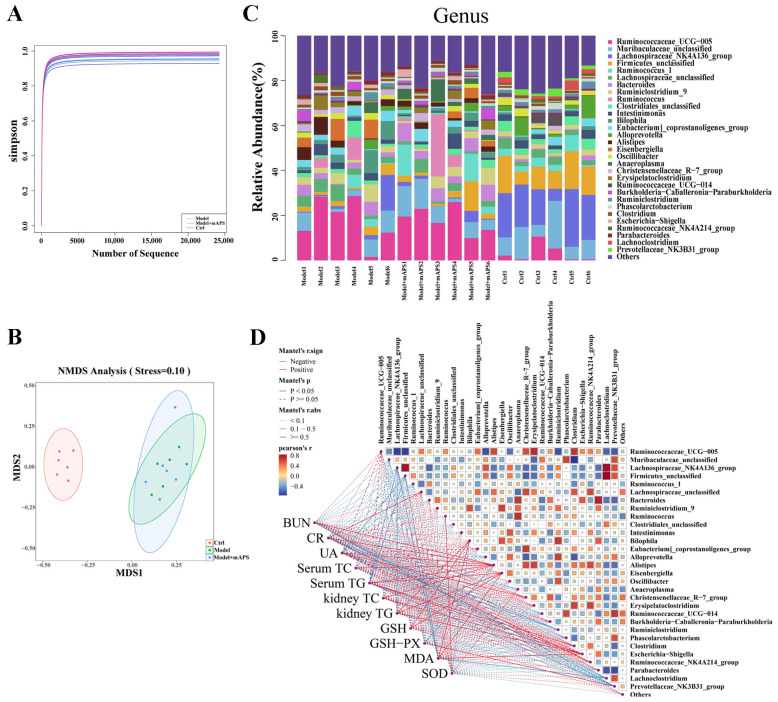
mAPS altered gut microbial composition (*n* = 6). (**A**) α-diversity analysis of gut microbiota based on Simpson indexes. (**B**) β-diversity analysis of gut microbiota based on NMDS. (**C**) Composition of bacteria Genus levels. (**D**) Spearman correlation between the genus levels of gut microbiota and biochemical parameters.

In this study, the 16S rDNA sequencing technique was applied to study the composition of fecal microbiota in different groups. As shown in Figure 9A, the shape of the Simpson’s exponential curve indicated that the depth of sequencing was sufficient. Non-Metric Multi-Dimensional Scaling (NMDS) analysis showed that the microbiota of the Model group and the Control group were clearly separated. There is no significant difference in the community composition of the gut microbiota between the Model and Model + mAPS rats (Figure 9B).

In the Control group, *Muribaculaceae*, *Ruminococcaceae_UCG-005*, and *Lachnospiraceae_NK4A136* accounted for 30% at the genus level (Top30). Compared with the rats in the Control group, the gut microbiome in the rats of the Model group exhibited significant changes, as characterized by the decreased abundance of *Muribaculaceae*, *Ruminococcus_1*, *Phascolarctobacterium*, and *Lachnoclostridium*, and elevated abundance of *Bilophila*, *Eubacterium*, *Alistipes*, *Eisenbergiella*, *Christensenellaceae_R-7*, and *Erysipelatoclostridium*. However, the abundance of these genera was restored with the administration of mAPS (Figure 9C).

Spearman’s correlation was used to analyze the relationship between the abundance of gut microbiota and the levels of biochemical parameters (Figure 9D). At the genus level, *Ruminococcaceae_UCG-005*, *Lachnospiraceae*, and *Bilophila* were positively correlated with the levels of BUN, CR, UA, TC, and TG. Meanwhile, *Muribaculaceae*, *Lachnospiraceae_NK4A136*, *Ruminococcus_1*, and *Clostridiales* presented a negative correlation with the levels of BUN, CR, UA, TC, and TG. The relationship was altered after mAPS treatment. Overall, these results indicate that the alteration in the gut microbiome following mAPS treatment resulted in a reduction in lipid accumulation and kidney injury in the model rats to some degree.

### 2.10. mAPS Improved Colon Injury in HSHFD/STZ-Induced Diabetic Rats

**Figure 10:**
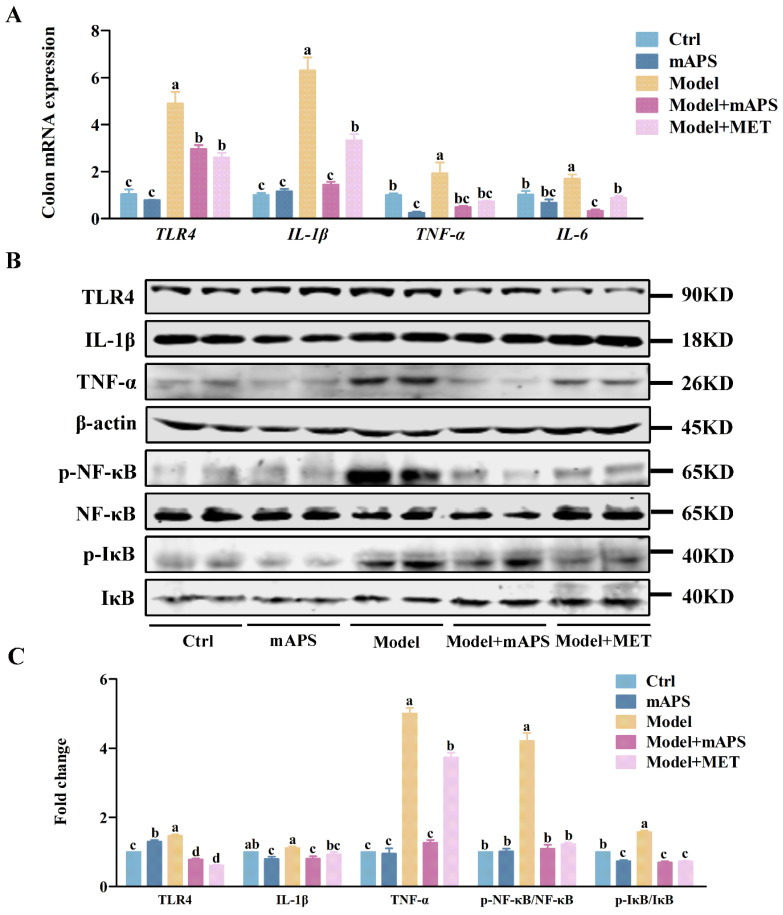
The effect of mAPS on the TLR4/NF-κB pathway in the colon. (**A**) The mRNA expression of *TLR4*, *IL-1β*, *TNF-α*, and *IL-6* in the colon. (**B**) The protein expression of TLR4, IL-1β, TNF-α, p-NF-κB, and p-IκB in the colon. (**C**) The fold change of TLR4, IL-1β, TNF-α, p-NF-κB, and p-IκB. Data are expressed as mean ± SD. Data with different letters are significantly different (*p* < 0.05).

An imbalance in gut microbiota is strongly linked to inflammation in the gut. Therefore, we detected a series of inflammatory cytokines in the colon in different groups of rats. As shown in Figure 10A, quantitative-PCR results indicated that the colonic *TLR4*, *IL-1β*, *TNF-α*, and *IL-6* mRNA levels of the model rats were significantly elevated compared to those in the control rats (*p* < 0.05). mAPS treatment significantly lowered these mRNA levels (*p* < 0.05). Furthermore, Western blotting results demonstrated that the protein expression of TLR4, IL-1β, TNF-α, p-NF-κB, and p-IκB in the Model group was significantly higher compared to that in the Control group (*p* < 0.05). Importantly, the administration of mAPS significantly reduced these elevated protein levels in the model rats. (Figure 10B,C, *p* < 0.05).

## 3. Discussion

Although the antihyperglycemic effects of mAPS in diabetic animal models have been reported, it remains unclear whether mAPS could improve DN. In this study, we found that mAPS had a therapeutic effect on DN induced by HSHFD and STZ. This effect is mediated through mechanisms involving the antioxidant Nrf-2/Keap-1 signaling pathway, the TLR4/NF-κB signaling pathway, the TGF-β/Smad signaling pathway, and gut microbiota modulation.

Firstly, the HFHSD/STZ-treated SD rats with fasting blood glucose consistently greater than 11.1 mmol/L were considered successful models of T2DM. The experimental findings revealed that SD rats exposed to HFHSD/STZ exhibited a marked elevation in the serum levels of BUN, SCR, and UA, indicator characteristics of diabetic nephropathy (DN), aligning with the observations reported in Chen’s study [25]. Notably, mAPS supplementation significantly reversed this trend and alleviated the symptoms of increased drinking, eating, urination, and weight loss in the HFHSD/STZ rats. Furthermore, histological examination demonstrated that mAPS ameliorated the structural injury of the colon and kidney. The results obtained suggest that mAPS exhibited a mitigating effect on the development of DN induced by HFHSD/STZ.

Hyperglycemia-induced oxidative stress stands as one of the primary pathogenic mechanisms underlying diabetic nephropathy (DN). Chen et al. [26] found that Astragalus polysaccharides have strong antioxidant activity, which can improve insulin resistance, attenuate oxidative stress, and alleviate liver injury. Similarly, Ma et al. [27] and Wang et al. [28] found that APS exhibited strong antioxidant activities both in vivo and in vitro, supporting the potential role of APS in alleviating oxidative stress. The results obtained in our study showed that mAPS significantly reduced the MDA levels while significantly increased the activities of GSH, GSH-PX, and SOD in the DN rats.

The Nrf-2/Keap-1 signaling pathway is regarded as a potential therapeutic target for mitigating oxidative stress implicated in a multitude of diseases [29,30,31]. Under normal physiological conditions, the transcription factor Nrf-2 remains inactive and sequestered in the cytoplasm through its binding to Keap-1 [32]. Oxidative stress weakens Keap-1′s inhibition, leading to the release of Nrf-2. This allows Nrf-2 to translocate into the nucleus and activate a suite of antioxidative stress genes, such as GCLC, NQO1, HO-1, and SOD. Consequently, this process shields the cell from oxidative damage [33]. To explore the potential antioxidant mechanism of mAPS in DN, we conducted a thorough examination of the expression of Nrf-2-targeted genes. Our results demonstrated that a four week administration of mAPS significantly upregulated the expression levels of Nrf-2, GCLC, NQO1, HO-1, and SOD, while concurrently downregulating the expression levels of Keap-1. These findings indicate a potential stimulatory effect of mAPS on the renal Nrf-2/Keap1 signaling pathway in the model rats. Our findings align with a prior study, which reported that APS ameliorates cognitive impairment and β-amyloid accumulation in mice by activating the Nrf-2/Keap1 pathway [34]. Thus, the robust nephroprotective properties of mAPS against oxidative stress hold significant therapeutic promise for the treatment of DN.

In addition to oxidative stress, inflammation also plays a pivotal role in the pathogenesis of DN [3,35]. In conjunction with oxidative stress-induced tissue damage, the release of reactive oxygen species (ROS) triggers the accumulation of inflammatory cells, leading to the production of inflammatory mediators that collectively exacerbate renal injury in DN [36]. The mechanism of the inflammatory response is the overactivation of the TLR4/NF-κB signaling pathway. The NF-κB family of nuclear transcription factors is vital for processes like immune-inflammatory responses, oxidative stress, and programmed cell death [37]. Consequently, modulating inflammation has emerged as a promising strategy to delay the progression of DN [38,39]. Our research demonstrated that the administration of mAPS also alleviated inflammation in the DN rats. The mechanism underlying this phenomenon may involve the regulation and modulation of the TLR4/NF-κB signaling pathway.

Emerging evidence suggests that kidney fibrosis is the final common pathway for most chronic progressive kidney diseases, including DN [40]. TGF-β signaling occupies a pivotal position in renal fibrogenesis, driving the transformation of fibroblasts into myofibroblasts and stimulating the excessive production of the extracellular matrix (ECM) [41]. When the TGF-β ligand binds to its receptor TGFβR, it triggers a cascade of downstream signaling events, including the activation of Smad proteins [42]. The Masson staining results from our study showed an increase in collagen deposition in the kidney of the model rats, which is in agreement with the observations made by Li et al. [43] in their study on DN. Furthermore, mAPS supplementation not only decreased the collagen content in the kidney of DN rats but also modulated the TGF-β-TGFβR-Smad4 signaling pathway. However, the detection of collagen production in the serum was not feasible due to the insufficient availability of serum samples.

Accumulating evidence indicates that gut microbiota play a significant role in the pathobiology of DN [44,45,46,47]. Our study found that the abundance of *Muribaculaceae*, *Ruminococcus_1*, and *Phascolarctobacterium* was reduced in the DN rats, and supplementation with mAPS restored the abundance of these bacteria. Smith et al. [48] confirmed that *Muribaculaceae* alleviates DM by modulating the host’s energy balance and glucose metabolism via the production of short-chain fatty acids (SCFAs). Studies have shown that *Ruminococcus* belongs to the *Peptostreptococcaceae* family and exhibits a remarkable capacity for polysaccharide degradation [49]. Meanwhile, our studies have shown that the abundance of *Muribaculaceae*, *Ruminococcus_1*, and *Phascolarctobacterium* are negatively correlated with the BUN, CR, UA, TC, and TG. Additionally, the abundance of *Bilophila* and *Alistipes* are positively correlated with BUN, CR, UA, TC, and TG. Reports from Natividad et al. [50] suggest that *Bilophila* interacts synergistically with a high-fat diet (HFD), leading to dysfunction in the intestinal barrier and bile acid metabolism. This interaction exacerbates impairments in glucose metabolism and hepatic steatosis. Zeng et al. [51] also validated *Alistipes* as a gut microbiota marker in obese patients. The Model + mAPS group exhibited a significantly reduced abundance of *Bilophila* and *Alistipes* compared to the Model group, suggesting that mAPS has the potential to alleviate gut microbiota disorders caused by diabetes mellitus. The above results suggest that alterations in the gut microbiota might be closely related to T2DM and DN. Therefore, we can further investigate the antidiabetic nephropathy (DN) activity of mAPS from the perspective of its effects on the gut microbiota. However, this study has limitations in terms of understanding the role of microbiota changes in the antidiabetic nephropathy (DN) effect of mAPS. It is imperative to conduct further research using antibiotics for microbiota clearance or to utilize germ-free mice to more accurately assess the modulation of the microbiota.

PPARγ is an important target in the metabolic and inflammatory processes of diabetes [52,53,54]. We detected the expression of PPARγ in the liver of diabetic rats. Compared with the Model group, the mRNA and protein levels of PPARγ were significantly increased after treatment with mAPS and MET (Figure S1). However, its expression was not detected in the kidney. Future studies should consider incorporating PPARγ inhibitors or activators to further explore their potential relationship with the progression of DN and the associated therapeutic effects.

## 4. Materials and Methods

### 4.1. Materials

Our previous study [24,55] reported the extraction and characterization of *Astragalus mongholicus* polysaccharides (mAPS). Streptozotocin (STZ) (S0130) was purchased from Sigma (New Mexico, NM, USA). Metformin (MET) was sourced from Meilun Biotechnology Co., Ltd. (Dalian, China). Regular diet (RD) and the high-sugar and high-fat diet (HSHFD) (main components: 24% fat, 24% protein, 41% carbohydrate) were purchased from Xiaoshu Youtai (Beijing, China). Antibodies against NLRP3 (#15101), IL-1β (#12242), NF-κB (#8242), IκB (#4812), phospho-NF-κB (#3033), and phospho-IκB (#2859) were obtained from Cell Signaling Technology (Danvers, MA, USA). Antibodies against Nrf-2 (ab31163), GCLC (ab190685), NQO1 (ab34173), HO-1 (ab13243), IKKα + IKKβ (ab194528), and GAPDH (ab8245) were obtained from Abcam (Cambridge, UK). Antibodies against TGFβR (PTM-5773) and Smad4 (PTM-5449) were purchased from PTM BIO (Hangzhou, China). Anti-TLR4 (WL00196), anti-SOD2 (WL0530a), anti-KEAP1 (WL03285), anti-TGFβ (WL02193), anti-α-Tubulin (WL02296), and anti-β-actin (WL01372) were purchased from Wan Lei Biology (Shenyang, China). IR Dye 800 CW IgG (H + L) was obtained from LI-COR Biosciences (Nebraska, NE, USA).

### 4.2. Animals and Experimental Design

A total of 30 SPF male SD rats (6–8 weeks, 200 ± 20 g) were obtained from Sibeifu Biotechnology Co., Ltd. (Beijing, China) and fed under the conditions of 25 ± 2 °C, 50 ± 5% humidity, and a 12/12 h light and dark cycle. The treatment of experimental animals conforms to the regulations of the Ethics Committee of Inner Mongolia Agricultural University (No: NND2021092, Date: 19 July 2021).

After one week of acclimatization, the rats were randomly divided into five groups (*n* = 6 per group): the Control group, the mAPS group, the Model group, the Model + mAPS group, and the Model + MET group. The Control and the mAPS groups were fed a regular diet, while the other groups received a high-sugar and high-fat (HSHFD) diet for four weeks. Streptozotocin (STZ) was then injected intraperitoneally (35 mg/kg) to induce diabetes. Rats in the Control and the mAPS group were injected intraperitoneally with a citrate buffer solution. A fasting blood glucose (FBG) level exceeding 11.1 mmol/L indicated the successful establishment of the model [56]. After model establishment, the mAPS and the Model + mAPS group were orally gavaged with 200 mg/kg mAPS. The Model + MET group was orally gavaged with 300 mg/kg metformin (MET). The dosage levels for mAPS and MET were chosen based on our previous study [24]. The treatment lasted 4 weeks. Four weeks later, all the rats were fasted for 12 h and then anesthetized through an intraperitoneal injection using a 1% pentobarbital sodium solution at a dose of 0.17 mL/100 g. After the collection of feces and blood samples, the rats were euthanized by cervical dislocation. The weight of the liver, kidney, and colon was measured. The colon and kidney sections were fixed with 4% paraformaldehyde, whereas the rest of the tissues were swiftly immersed in liquid nitrogen and then stored at −80 °C until further processing.

### 4.3. Biochemical Analysis

FBG levels in different groups were measured at designated time points using a blood glucose meter (i-SENS, Incheon, Republic of Korea). The collected serum was assayed according to the procedure of TC, TG, LDL, HDL, Cr, BUN, UA, MDA, SOD, GSH, and GSH-PX assay kits to determine the index levels. Kidney tissues were also collected and assayed according to the procedure of TC and TG assay kits from Jiancheng Biotechnology Co., Ltd. (Nanjing, China) to determine the index levels.

### 4.4. Histological Analysis

After 24 h immersion in 4% paraformaldehyde/PBS, the samples of the kidney and colon were then dehydrated by using graded alcohol, cleared by using xylene, and embedded in paraffin. The tissue sections (5 μm) were stained with hematoxylin and eosin. According to the International standard for terminology and diagnostic criteria of pathological changes in rats and mice (INHAND) [57], each tissue section was carefully observed under a microscope, and histological changes were recorded. A four-point grading system was employed to evaluate the extent of tissue lesions, with the following designations: a score of 0 signified the absence of lesions, falling within normal limits; a score of 1 denoted mild lesion or a small number of lesions; a score of 2 indicated moderate lesions; and a score of 3 represented severe or widespread lesions. The fundamental pathological alterations, including congestion, edema, degeneration, necrosis, hyperplasia, fibrosis, inflammation, and other relevant changes observed in the tissue section, were individually scored. The ultimate score was then derived by aggregating these individual scores. A Masson staining kit was used to observe the degree of kidney injury and fibrosis (technical support was provided by Wuhan Service Biotechnology, Wuhan, China).

### 4.5. Quantitative PCR

The RNA of fresh colon and kidney tissue was extracted with TRIzol reagent, and the RNA quality was assessed using the NanoDrop2000 Spectrophotometer (Thermo Fisher Scientific, Waltham, MA, USA). The extracted RNA (1 μg) was reverse transcribed into cDNA. The primers were ordered from Sangon Biotech (Shanghai, China). The primer sequences used for the experiments are listed in Table 1. The mRNA expression of each gene was calculated as the difference in the threshold cycle (CT). The relative expression levels of each gene were analyzed by using the 2^−ΔΔCt^ method.

### 4.6. Western Blotting

Proteins from the nucleus and cytoplasm of colon and kidney tissues were extracted using the Nucleoprotein Extraction Kit (Sangon Bioengineering, Shanghai, China). Protein concentrations were quantified using a BCA (bicinchoninic acid) assay kit (Sangon Bioengineering, Shanghai, China). The extracted proteins underwent separation via SDS-PAGE electrophoresis and were subsequently transferred onto PVDF membranes sourced from Sigma–Aldrich (Shanghai, China). The membranes were blocked using 5% skimmed milk powder before incubation with primary antibodies (1/1000 dilution) at 4 °C overnight. After washing five times with Tris-buffered saline + Tween 20 (TBST), the PVDF membranes were then incubated with Goat anti-Rabbit IgG HandL (IRDye^®^ 800 CW, 1/10,000 dilution) for 1.5 h. The expression of these proteins was detected using the Odyssey infrared laser scanning imager (LI-COR).

### 4.7. 16S rDNA Gene Sequencing

Total DNA was extracted from gut contents and stool samples using E.Z.N.A. ^®^Stool DNA Kit. The V3-V4 variable region was amplified by polymerase chain reaction (PCR) using primers 341F (5′-CCTACGGGNGGCWGCAG-3′) and 805R (5′-GACTACHVGGGTATCTAATCC-3′). After quantification, purified PCR products of equal amounts were subjected to paired-end sequencing on the Illumina NovaSeq platform (Illumina, San Diego, CA, USA). Sequencing was conducted in accordance with the guidelines provided by Lc-Bio Technologies (Hangzhou, China).

### 4.8. Data Analysis

The R language was used to analyze and visualize the 16S rDNA sequencing results. GraphPad Prism 8.4 was used to perform analysis and graphing of data. The results were analyzed using SPSS 21 software. Pairwise comparisons at each time point were conducted using one-way ANOVA. Multiple comparisons were conducted using Duncan’s multiple range test. The experimental data were presented as the mean ± standard deviation (SD). Statistical significance between groups in the experimental data was determined at *p* < 0.05.

## 5. Conclusions

In summary, the findings of our study demonstrate that mAPS regulate oxidative stress, inflammation, and gut microbiota to further improve renal function in DN model rats. These results underscore the potential clinical applicability of mAPS as a therapeutic strategy for DN.

## Figures and Tables

**Table 1:** Sequences of primers used for Quantitative PCR.

The Name of The Primer	Primer Sequences
Nrf-2 sense	CCCAGCAGGACATGGATTTG
Nrf-2 antisense	TTTGGGAATGTGGGCAACCT
NQO-1 sense	CAGCGGCTCCATGTACT
NQO-1 antisense	GACCTGGAAGCCACAGAAG
SOD-2 sense	TTGGCTTCAATAAGGAGCAAG
SOD-2 antisense	ACACATCAATCCCCAGCAGT
Keap-1 sense	TAGCCTCCATGAAGCATCGC
Keap-1 antisense	GTCAAGCGGGTCACTTCACT
HO-1 sense	ACATTGAGCTGTTTGAGGAGC
HO-1 antisense	CTGAGTGTGAGGACCCATCG
IL-6 sense	CCACCAGGAACGAAAGTCAAC
IL-6 antisense	TTGCGGAGAGAAACTTCATAGCT
IL-10 sense	GCCCAGAAATCAAGGAGCATT
IL-10 antisense	CAGCTGTATCCAGAGGGTCTTCA
IL-1β sense	TGCTGTCTGACCCATGTGAG
IL-1β antisense	GTCGTTGCTTGTCTCTCCTTG
TNF-α sense	ATCGGTCCCAACAAGGAGGA
TNF-α antisense	TCCGCTTGGTGGTTTGCTAC
TLR4 sense	TGAGGACTGGGTGAGAAATGAGC
TLR4 antisense	CTGCCATGTTTTGAGCAATCTCAT
NLRP3 sense	GTGGAGATCCTAGGTTTCTCTG
NLRP3 antisense	CAGGATCTCATTCTCTTGGATC
TGF-β sense	CAACAATTCCTGGCGTTACCT
TGF-β antisense	AAAGCCCTGTATTCCGTCTCC
Actin sense	CGCGAGTACAACCTTCTTGC
Actin antisense	ATACCCACCATCACACCCTGG

## Data Availability

Data are contained within the article and Supplementary Materials.

## Supplementary Materials

The following supporting information can be downloaded at: https://www.mdpi.com/article/10.3390/ijms26041470/s1.

## Abbreviations

BUN	Blood urea nitrogen
CR	Creatinine
DM	Diabetes mellitus
DN	Diabetic nephropathy
FBG	Fasting blood glucose
GCLC	Glutamate-cysteine ligase catalytic subunit
GSH	Glutathione
GSH-PX	Glutathione peroxidase
HDL	High-density lipoprotein
HE	Hematoxylin and eosin
HO-1	Heme oxygenase 1
HSHFD	High-sugar and high-fat diet
IκB	Inhibitor of kappa B
IKKα + IKKβ	IκB kinase alpha + IκB kinase beta
IL-10	Interleukin-10
IL-1β	Interleukin-1beta
IL-6	Interleukin-6
Keap1	Kelch-like ECH-associated protein 1
LDL	Low-density lipoprotein
LPS	Lipopolysaccharide
mAPS	Astragalus mongholicus polysaccharides
MDA	Malondialdehyde
MET	Metformin
NF-κB	Nuclear factor kappa-B
NLRP3	NOD-like receptor protein 3
NQO1	NAD(P)H quinone dehydrogenase 1
Nrf-2	Nuclear factor erythroid 2-related factor 2
PCR	Polymerase chain reaction
Smad4	Smad family member 4
SOD	Superoxide dismutase
STZ	Streptozotocin
TC	Total cholesterol
TG	Triglyceride
TGF-β	Transforming growth factor beta
TGFβR	Transforming growth factor beta-receptor
TLR4	Toll-like receptor 4
TNF-α	Tumor necrosis factor-alpha
UA	Uric acid
ZO-1	Zona occludens 1
