# Supplementary figures and images for: Astragalus Mongholicus Polysaccharides Alleviate Kidney Injury in Rats with Type 2 Diabetes Through Modulation of Oxidation, Inflammation, and Gut Microbiota
Source: Int J Mol Sci. 2025 Feb 10;26(4):1470. doi: 10.3390/ijms26041470 (PMC11855448; doi:10.3390/ijms26041470)

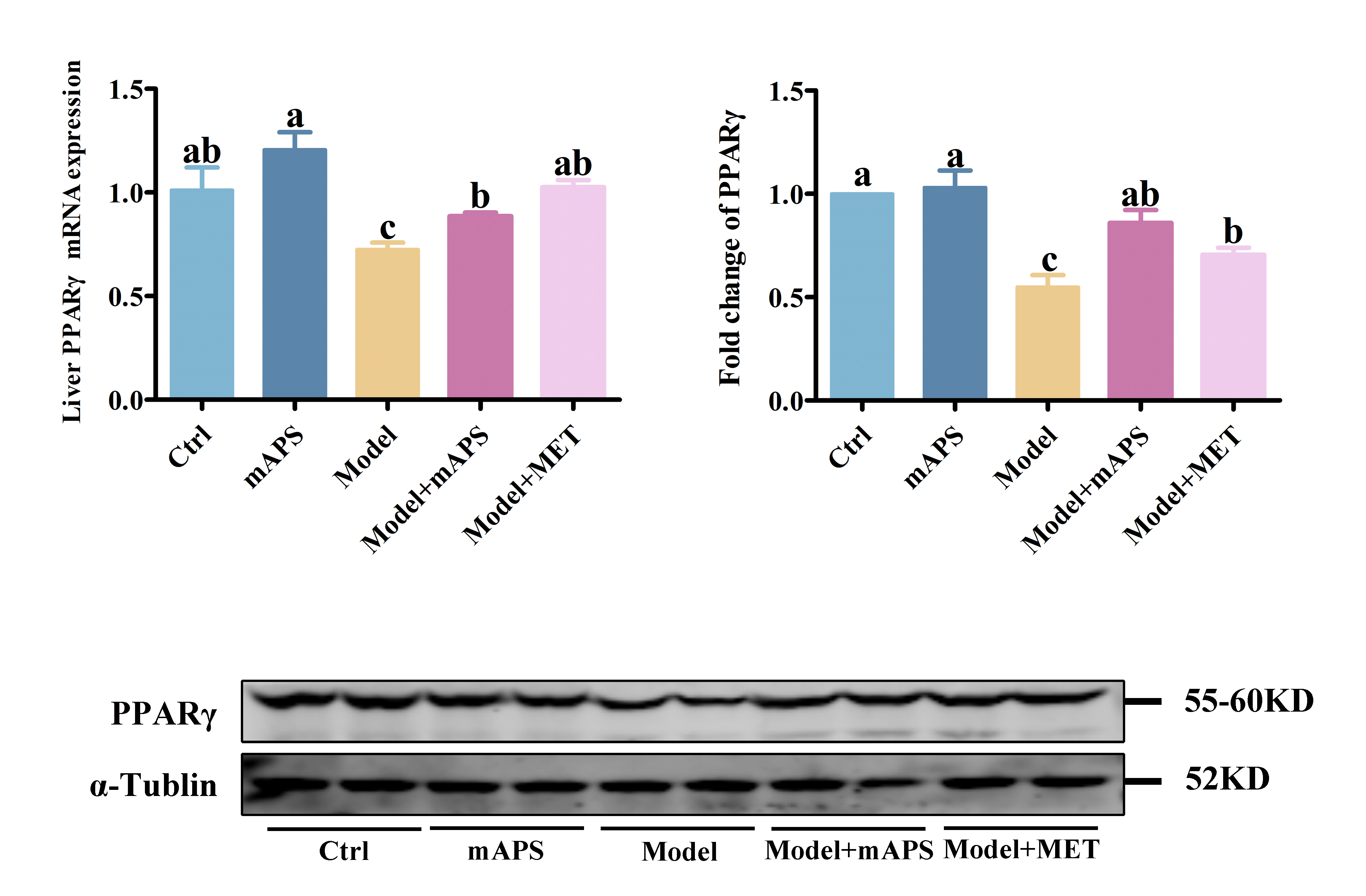

Supplement: Supplementary file 1 [file ijms-26-01470-s001.zip › ijms-3453828-supplementary.tif]
